# Evaluation of Diagnostic Accuracy of Eight Commercial Assays for the Detection of Measles Virus-Specific IgM Antibodies

**DOI:** 10.1128/JCM.03161-20

**Published:** 2021-05-19

**Authors:** Joanne Hiebert, Vanessa Zubach, Carmen L. Charlton, Jayne Fenton, Graham A. Tipples, Kevin Fonseca, Alberto Severini

**Affiliations:** a Viral Exanthemata and STD Section, National Microbiology Laboratory, Public Health Agency of Canada, Winnipeg, Manitoba, Canada; b Public Health Laboratory, Alberta Precision Laboratories, Edmonton, Alberta, Canada; c Department of Laboratory Medicine and Pathology, University of Alberta, Edmonton, Alberta, Canada; d Li Ka Shing Institute of Virology, University of Alberta, Edmonton, Alberta, Canada; e Department of Medical Microbiology and Immunology, University of Alberta, Edmonton, Alberta, Canada; f Department of Microbiology, Immunology, and Infectious Disease, University of Calgary, Calgary, Alberta, Canada; g Department of Medical Microbiology and Infectious Diseases, Faculty of Health Sciences, University of Manitoba, Winnipeg, Manitoba, Canada; Rhode Island Hospital

**Keywords:** measles IgM serology, ELISA, EIA, CLIA, sensitivity, specificity, IgM, immunoserology, kit evaluation, measles

## Abstract

The World Health Organization (WHO) has targeted measles for global eradication through mass immunization. For effective monitoring of eradication targets, high-quality surveillance is needed. The detection of IgM antibodies, specific to the measles virus, with the use of commercial enzyme-linked immunosorbent assays (ELISA or EIA) is broadly used within the WHO global measles and rubella laboratory network for laboratory confirmation, and in particular, ELISA kits manufactured by Siemens (Enzygnost kits) have been primarily used. Spurred by the discontinuation of these kits, this study aims to report on the clinical sensitivity and specificity of comparable commercial ELISA kits and one automated chemiluminescent immunoassay (CLIA) method. A panel of 239 serum samples was assembled that included sera from confirmed measles cases (*n* = 50) and probable post-MMR vaccine response (*n* = 2). Measles-negative sera (*n* = 187) were collected from individuals presenting with other fever and rash illnesses. A total of 7 ELISA kits (Euroimmun native antigens and recombinant nucleoprotein, IBL, Clin-Tech Microimmune, NovaTec NovaLisa, Serion, and Siemens Enzygnost) and one CLIA method (DiaSorin LIAISON XL) were evaluated. The ELISA kits included two IgM capture methods and five indirect methods. Calculated sensitivities and specificities ranged from 75.0% to 98.1% and 86.6% to 99.5%, respectively. The parvovirus B19 IgM positive sera were noted to cause false-positive results, particularly for the ELISA kits from Serion and NovaLisa; specificities for this subset of samples ranged from 51.4% to 100.0%. The capture IgM ELISA methods provided the best combination of sensitivity and specificity.

## INTRODUCTION

Measles, once typically a childhood illness characterized by high fever and rash, is caused by infection with the measles virus (MeV) and can be prevented with an effective vaccine that has been available since the 1960s (1). Endemic measles circulation has been interrupted in most countries of the Americas, including Canada, and in other regions of the world (2, 3). However, due to the highly infectious nature of the virus and ongoing circulation elsewhere, outbreaks can and do still occur (4–8). The largest outbreak of measles in Canada since elimination occurred in 2011 and totaled 725 cases in a province with high vaccination coverage (4).

The World Health Organization (WHO) has targeted measles for global eradication through mass immunization (9). For effective surveillance of eradication targets, high-quality surveillance is needed, including accurate laboratory diagnostic methods. The detection of IgM antibodies, specific to the measles virus, with the use of commercial, 96-well plate-based enzyme-linked immunosorbent assays (ELISA or EIA) is broadly used within the WHO global measles and rubella laboratory network (10). Previous evaluation studies led to the broad adoption of the Enzygnost kit from Siemens as the method of choice (10–13). As this kit has recently been discontinued (14), independently validated replacements are urgently needed. Using residual sera collected from confirmed cases during measles outbreaks, this study aimed to evaluate ELISA methods identified by network laboratories that were similar to the Enzygnost methodology and suitable for use in settings with limited automation. One automated chemiluminescent method was included for its applicability to laboratories with access to automated instrumentation.

## MATERIALS AND METHODS

### Panel sample set.

The study was conducted retrospectively and employed anonymized residual sera that had been received either at the National Microbiology Laboratory (NML) or the Alberta Public Health Laboratory (ProvLab) for serological testing (convenience sampling). Sera from 68 laboratory-confirmed cases that met the national case definition for measles by local public health authorities and collected during measles outbreaks were sent to the NML in 2013 for confirmatory measles serology. All sera were reported by the referring laboratory to be anti-measles IgM positive or equivocal. The residual volume of all specimens was insufficient for use on the DiaSorin LIAISON XL method, which requires at least 170 μl; thus, the sera were pooled to create a total of 49 specimens with volumes of 170 to 190 μl (Table 1). The pooling scheme was such that the sera with the highest volumes were identified (estimated to be 110 to >150 μl), and to each of those, a second serum specimen was added (median, 35 μl; range, 30 to 100 μl) (*n* = 43). For 6 specimens, an additional third serum was added, due to insufficient volume after the initial top-up. An additional specimen, sourced from a company that supplies sera with known acute measles infection status to proficiency panel providers was included in the confirmed measles panel (sample number 357006; GBD [Gesellschaft für Biotechnologische Diagnostik mbH], Berlin, Germany).

**TABLE 1:** Characteristics of the serum samples included in this study

Sample group	Source	No. of patients	No. of serum samples	Median age, yrs (range)
Measles sera
Confirmed measles cases	Sera from measles outbreaks	68	49 (pooled)	15 (1–53)
Confirmed measles cases	Commercial serum supplier; acute measles infection	Unknown	1	Unknown
Probable post-MMR reactions	Sera submitted for suspected primary HHV-6	2	2	1 (1–1)
Total measles sera	52	14 (1–53)
Non-measles sera
Chikungunya IgM positive	Sera submitted for suspected chikungunya infection	4	4	41 (40–56)
Dengue IgM positive	Sera submitted for suspected dengue infection	Unknown	34	Unknown
Fever + rash of unknown etiology	Sera submitted for suspected primary HHV-6, HHV-6 IgM negative, and IgG positive	37	37	7 (0–67)
HHV-6 IgM positive	Sera submitted for suspected primary HHV-6	22	22	1 (0–3)
HHV-6 PCR and IgM positive	Sera submitted for suspected primary HHV-6	16	16	0.5 (0–2)
Parvovirus B19 IgM positive	Sera submitted for suspected parvovirus B19 infection	35	35	36 (7–50)
Rubella IgM positive	Leftover sera from proficiency panel program, includes commercial sera	Unknown	36	Unknown
Zika IgM positive	Sera submitted for suspected Zika virus infection	3	3	30 (26–52)
Total non-measles sera	187	7 (0–67)
Total panel sera	239	11 (0–67)

A total of 153 residual clinical sera that were confirmed to be IgM positive for other fever- and rash-causing viruses were included in the panel (Table 1). Agents included were chikungunya (*n* = 4), dengue (*n* = 34, 3 of which were pooled in a similar manner as the measles sera), human herpesvirus 6 (HHV-6) (*n* = 40, 16 of which were also PCR positive), parvovirus B19 (*n* = 35), and Zika virus (*n* = 3). Thirty-seven sera from clinical cases with fever and rash, as recorded on the test requisition, and referred for HHV-6 serology with HHV-6 IgM-negative/IgG-positive results were included. The sera for which collection dates were known were collected between 2001 and 2015. An additional 36 sera, available as part of the inventory of the NML’s rubella serology proficiency panel program, were included. These were archival sera sourced from a variety of suppliers, including commercial rubella IgM-positive controls.

The final panel (*n* = 239) was assembled, randomized, and blinded at the NML. The panel was frozen and sent to the Alberta provincial laboratory for testing on the automated platform DiaSorin LIAISON XL. Upon completion, the panel was refrozen and returned to the NML for all plate-based ELISAs.

### DiaSorin LIAISON XL chemiluminescent assay.

The LIAISON XL measles IgM assay is a high-volume commercial platform which uses viral recombinant antigen in a direct IgM microcapture assay. A single technologist at the Public Health Laboratory in Alberta ran all samples according to the manufacturer’s instructions. Calibrators and instrument controls were within range for all specimens tested. An external positive control was included in all assays in triplicate or singly as volume permitted.

### ELISA serological methods.

The following commercial ELISA kits for the detection of measles IgM were included in the evaluation: Euroimmun (native antigens and recombinant nucleoprotein), IBL, Microimmune, NovaLisa, Serion, and Enzygnost (details provided in Table 2). All methods were performed by a single technician at the NML according to the manufacturer’s instructions for use (IFU) provided in the kits, except for the volume of serum used if it exceeded 5 μl in the IFU, due to the limited volume available. For those kits (Enzygnost, Euroimmun, Euroimmun NP, NovaLisa, and Serion), the volume of serum used was reduced by half and the volumes of dilution buffer used were adjusted accordingly to maintain the dilution ratio given in the IFU. Specifically, for the Enzygnost kit, 10 μl of serum was diluted with 200 μl of sample buffer, except for the kit controls, which were diluted as instructed (20 μl diluted with 400 μl sample buffer). For the Euroimmun (both kits), NovaLisa, and Serion kits, 5 μl of serum was used rather than the 10 μl in the IFU and diluted with half the volume of the dilution buffer (500 μl) listed in the IFU. The same external positive control was included in all ELISA methods and all test plates, in duplicate or singly as it became depleted (specifically, on the Euroimmun NP kit). Washing steps were automated on a BioTek 50TS 96-well plate washer. Temperatures (room temperature and 37°C incubator) were verified with calibrated thermometers to be within the limits given in the kit IFU prior to performing the tests. Optical densities (ODs) were read as per the IFU with a Tecan Sunrise microplate absorbance reader. Optical density data were exported to a Microsoft Excel 2016 file and then copied into custom-made, verified Microsoft Excel 2016 templates where calculations and result determinations were automated. Test plate validation and specimen results were determined as per the manufacturers’ IFU. The Serion kit IFU included three possible methods of generating a qualitative result (activity calculator, OD range, and special case formula), and all methods were followed. Samples with equivocal results were repeated if advised in the IFU (Microimmune). All ELISA kits were tested in a single freeze-thaw cycle of the panel, with the exception of the repeats of equivocal results and the Euroimmun NP kit.

**TABLE 2:** Characteristics of the commercial kits for the detection of anti-measles IgM antibodies evaluated in this study^*a*^

Characteristic	Enzygnost (Siemens Healthcare Diagnostics Products GmbH, Marburg, Germany)	Euroimmun (Euroimmun Medizinische Labordianostika AG, Lübeck, Germany)	Euroimmun Nucleoprotein (Euroimmun Medizinische Labordianostika AG, Lübeck, Germany)	IBL (IBL International GmbH, Hamburg, Germany)	LIAISON XL (DiaSorin Saluggia, Vercelli, Italy)	Microimmune (Clin-Tech Ltd., Guildford, UK)	NovaLisa (NovaTec Immundiagnostica GmbH, Dietzenbach, Germany)	Serion Classic (Institut Virion\Serion GmbH, Würzburg, Germany)
Catalogue no.	OWLI15	EI 2610-9601 M	EI 2610-9601-4 M	RE57151	318820	MeVM010	MEAM0330	ESR102M
Lot no. evaluated	48580, 48747	E190201AG	E191120BR	IMEM138	174022	K86-153-12	MEAM-106	SGI.Ef
Version no. of IFU^*b*^ evaluated	2015-05	01/08/2013	09/10/2013	42345	EN-200/007-028, 05-2016	K50p201709	04062019	V 102.14
European regulatory status^*c*^	CE IVD	CE IVD	CE IVD	CE IVD	CE IVD	CE IVD	RUO	CE IVD
Health Canada clearance	Yes	Yes	Yes	Yes	Yes	No	No	No
U.S. FDA clearance	No	No	No	No	No	No	No	No
Method description^*e*^	Indirect ELISA	Indirect ELISA	Indirect ELISA	IgM capture (anti-IgM antibody coated wells)	Automated IgM capture CLIA^*d*^	IgM capture (anti-IgM antibody coated wells)	Indirect ELISA	Indirect ELISA
Antigen	Paired whole virus and control (cellular) antigen wells	Whole virus	Recombinant measles nucleoprotein	Whole virus	Recombinant measles nucleoprotein	Recombinant measles nucleoprotein	Whole virus	Not specified
Use of RF^*f*^ absorbent	Yes, separate incubation step	Yes, separate incubation step	Yes, separate incubation step	No	No	No	Yes but no additional incubation	Yes, separate incubation step
Incubation conditions	37°C, humidified	Room temp (18–25°C)	Room temp (18–25°C)	37°C	NA; completely automated	37°C, humidified	37°C	37°C, humidified
No. of reagents to prepare	4	1	1	2	0	2	1	2
Total incubation time	2 h, 45 min	1 h, 25 min	1 h, 25 min	2 h, 30 min	NA; completely automated	1 h, 40 min	1 h, 45 min	2 h, 15 min
Approximate total time	3 h	1 h, 40 min	1 h, 40 min	2 h, 45 min	45 min	2 h	2 h	2 h, 30 min
Maximum no. of samples per plate (per kit)	45 (90)	93 (93)	93 (93)	93 (93)	50	92 (92)	92 (92)	92 (92)
Shortest reagent shelf life once opened	2 mo (strips)	4 mo (strips)	4 mo (strips)	Same as kit expiry (∼1 yr)	8 wks on board	3 mo (strips)	Same as kit expiry (∼1 yr)	4 wks (strips)
Completeness of kit	Supplemental kit required (catalogue no. OUVP)	All reagents provided	All reagents provided	All reagents provided	Controls separate	All reagents provided	All reagents provided	RF-absorbent separate (catalogue no. Z200)
Serum vol	20 μl, as per the IFU; 10 μl was used	10 μl, as per the IFU; 5 μl was used	10 μl, as per the IFU; 5 μl was used	5 μl, as per the IFU	20 μl used with minimum 150 μl dead vol	5 μl, as per the IFU	10 μl, as per the IFU; 5 μl was used	10 μl, as per the IFU; 5 μl was used
Total cost per test sample for this study, USD	$5.44	$3.83	$3.37	$2.61	$2.94 (IgM kit only: no support materials)	$3.92	$2.16	$2.58

a NA, not applicable..

b IFU, instructions for use provided in the kit.

c CE, Conformitè Europëenne Mark (CE Mark); IVD, i*n vitro* diagnostic device; RUO, research use only.

d Chemiluminescent assay.

e ELISA, enzyme-linked immunosorbent assay.

f RF, rheumatoid factors.

### Treatment of equivocal results.

All methods included an indeterminate range where the result could not be categorized as either positive or negative. These were handled in two ways for assessment of test performance, an always wrong approach and a presumptive positive approach. In both scenarios, equivocal results with the non-measles sera were always considered positive. Thus, only one specificity value was calculated for each method. Sensitivity and accuracy were calculated using both approaches where the equivocal results for the measles sera were considered negative (“always wrong”) or positive (“presumptive positive”). (For the accuracy calculations, equivocal results with the non-measles sera were always considered positive.)

### Data analysis.

Microsoft Excel 2016 was used to compile results and calculate sensitivity, specificity, and accuracy values and their 95% confidence intervals (CI). Confidence intervals were calculated using the score method (Specifically, L [lower limit] = {2*np* + z2 – 1 – z √ [z2 – 2 – (1/*n*) + 4*p*(*n*q + 1)]}/2(*n* + z2) and U [upper limit] = {2*np* + z2 + 1 + z √ [z2 + 2 – (1/*n*) + 4*p*(*n*q - 1)]}/2(*n* + z2), where z = 1.96, *p* is the sensitivity or specificity, and q = 1 – *p*. If *p* = 1, then U = 1, since specificity and sensitivity cannot be >100%.) (15).

An online calculator was used to calculate the kappa measure of agreement and 95% confidence intervals (GraphPad, available at https://www.graphpad.com/quickcalcs/kappa1/).

GraphPad Prism 8.3.0 was used to generate box plots of the normalized output values for each method. Each ELISA method performed method-specific manipulations of the raw OD values to generate a test output value (which was then used to determine the qualitative result). The scale of the resulting output values was not comparable across methods, particularly for the Serion activity calculator method, which generated values in the thousands, while most other methods had values of <10. Therefore, the data for each method were normalized to 100 to facilitate their comparison. In Microsoft Excel 2016, the grouped measles and non-measles output values were sorted from highest to lowest value for each method. The highest value for each method was arbitrarily set to 100. To determine the normalized value for the remaining values, each value was divided by the highest value for the method and then multiplied by 100. The normalized values were used to generate the box plots.

## RESULTS

### Panel samples.

A panel of 239 sera was assembled that included 50 pooled specimens from confirmed cases of measles and 189 sera that were IgM positive for a number of other viruses (chikungunya, dengue, HHV-6 [roseola], parvovirus B19, rubella, Zika) or presented with fever and rash of unknown etiology (the “non-measles” panel) (Table 1). These viruses were chosen because they can also present with fever and rash symptoms and thus may be captured in measles testing algorithms, including IgM. This panel included, primarily as part of the roseola subset, a number of sera collected from individuals who were eligible to receive their first dose of measles-containing vaccine, which in Canada is recommended at 12 months of age and is combined with mumps and rubella (MMR) or mumps, rubella, and varicella (MMRV) (16). Thus, it was possible that some of the sera included in the non-measles panel were collected from recently vaccinated individuals. The assembled panel was also evaluated on 8 commercial ELISA kits for the detection of anti-rubella IgM, and rubella IgG avidity was determined (manuscript in preparation). The results were reviewed to determine and control for the likelihood of recent vaccination in the non-measles panel. Two of the roseola subset sera, collected from infants 1 year of age, had low rubella IgG avidity, and one was rubella IgM positive in 7 of 8 tested methods, while the second had equivocal or negative results. These two sera were also evaluated for the presence of anti-mumps IgM antibody (Euroimmun; catalogue number EI 2630-9601 M; data not shown), and both were positive. As a result, both sera were classified as probable post-MMR vaccine reactions for a total of 52 measles panel sera and 187 non-measles sera (Table 1).

### Kit characteristics.

Several characteristics, such as choice of antigen, completeness of the kit components, length of time needed for the test, incubation temperatures, and user-friendliness, of the seven commercial microplate-based ELISA kits that were included in the study were compared (Table 2). Two kits, from IBL and Microimmune, were IgM capture kits with anti-IgM antibody-coated wells, while the remaining five were indirect ELISAs. Of the indirect ELISAs, all but one used whole viral antigen; the remaining kit used recombinant nucleoprotein as the antigen. While most kits had comparable shelf life when unopened (approximately 1 year), once opened, the stability varied widely, with Serion having the shortest shelf life, of 4 weeks. Additional reagents had to be purchased separately for the Enzygnost and Serion kits—a supplemental kit (containing the wash buffer, substrate, and stop solution) and the rheumatoid factor (RF) absorbent, respectively. The method for result determination was clear for all kits, with the exception of the Serion kit, where the IFU included three possible methods for determining the result, without guidance on their application. Two of the three methods (the special case formula and OD range methods) were similar in that the positive and negative cutoff values were variable by test plate and depended on the OD values of the kit controls on the test plate. The special case formula used lot-specific constant values together with the test plate control OD values to generate test plate-specific positive and negative cutoff values, similar to the method used by Microimmune, while the OD range method used a choice of fixed cutoff values that was determined by the OD of the kit control on the test plate. The third method, the activity calculator, required the use of a complicated Excel template, obtained from Serion, that used 4-parameter logistic (4 PL) mathematical curve fitting to generate a quantitative value, in units/ml, that was converted to a qualitative result with lot-specific cutoffs. For this method, lot-specific curve parameters were provided which allowed the curve to be applied to the standard control, run in duplicate. The quantitative value for the specimens, run singly, was then interpolated.

### Assessment of measles IgM kit sensitivity.

The measles sera panel, consisting of 52 specimens (Table 1), was used to evaluate the sensitivity of the kits, calculated in two ways with equivocal results considered negative or positive. In both scenarios, most kits had sensitivities exceeding 90%, with the exception of the LIAISON XL system and the Euroimmun NP kit (Table 3). The reference method Enzygnost kit had a sensitivity of 94.2% when equivocals were considered negative, and three kits matched or exceeded this level, IBL, NovaLisa, and Serion. However, with a presumptive positive approach, the calculated sensitivity for the Enzygnost kit improved to 98.1%, which was not exceeded by any other kit. The Euroimmun kits (NP and whole antigen) had the lowest sensitivity at 75.0% and 78.8%, respectively, when equivocal results were considered negative, but these kits also had the highest number of equivocal results. Thus, when the equivocal results were considered positive, the calculated sensitivity improved to 86.5% and 90.4%, respectively. The best sensitivity was achieved with the Serion kit (98.1%; 95% CI, 88.4% to 99.9%), irrespective of result determination method and treatment of equivocal results. When equivocal results were considered negative, the Serion kit had a statistically significantly better sensitivity than that of the lowest-performing kit, the Euroimmun NP (75.0%; 95% CI, 60.8% to 85.5%) (Table 3).

**TABLE 3:** Results and calculated sensitivities, with equivocal results counted as either negative or positive, of the commercial methods for the detection of anti-measles IgM antibodies evaluated with the measles sera panel (*n* = 52)

Method	No. positive	No. equivocal	Sensitivity (%)^*a*^	95% CI (%)^*a*^	Sensitivity (%)^*b*^	95% CI (%)^*b*^
Enzygnost	49	2	94.2	83.1–98.5	98.1	88.4–99.9
Euroimmun	41	6	78.8	64.9–88.5	90.4	78.2–96.4
Euroimmun Nucleoprotein	39	6	75.0^*d*^	60.8–85.5	86.5	73.6–94.0
IBL	49	1	94.2	83.1–98.5	96.2	85.7–99.3
LIAISON XL	45	0	86.5	73.6–94.0	86.5	73.6–94.0
Microimmune	48	3	92.3	80.6–97.5	98.1	88.4–99.9
NovaLisa	49	0	94.2	83.1–98.5	94.2	83.1–98.5
Serion (activity calculator)^*c*^	51	0	98.1^*d*^	88.4–99.9	98.1	88.4–99.9
Serion (OD range)^*c*^	51	0	98.1^*d*^	88.4–99.9	98.1	88.4–99.9
Serion (special case formula)^*c*^	51	0	98.1^*d*^	88.4–99.9	98.1	88.4–99.9

a Specimens with equivocal results counted as negative.

b Specimens with equivocal results counted as positive.

c Three methods of sample result determination, using the single set of optical density data from the test plates, were provided in the manufacturer’s IFU. All three methods were evaluated.

d Significant difference (*P* < 0.05) between the most sensitive (Serion, all three result determination methods) and the least sensitive methods (Euroimmun NP) based on nonoverlapping 95% confidence intervals. This difference is only significant when equivocal results are counted as negative.

### Assessment of measles IgM kit specificity.

The results of testing with the non-measles sera panel, which consisted of 7 subsets of panels for a total of 187 specimens (Table 1), was used to calculate the specificity of the kits. Only unequivocally negative results were included in the specificity determination; equivocal results were included in the denominator (effectively counted as positive). The calculated specificities ranged from 86.6% (Serion, special case formula result determination method; 95% CI, 80.7% to 91.0%) to 99.5% (LIASION XL; 95% CI, 96.6% to 100%) (Table 4). The reference method Enzygnost kit had a specificity of 95.2% (95% CI, 90.8% to 97.6%), and five kits exceeded this level, LIAISON XL, Euroimmun, Euroimmun NP, IBL, and Microimmune. Three of these kits (LIAISON XL, Euroimmun NP, and Microimmune) had specificities that were statistically significantly better than those of the lowest-performing kits, NovaLisa and Serion (all three result determination methods). The remaining two (Euroimmun and IBL) were statistically significantly better than the Serion kit, but only when the special case formula result determination method was used. A difference, not statistically significant, was noted in the specificities calculated for the Serion kit depending on the method of result determination, with the OD range method resulting in the fewest false-positive or equivocal results (Table 4).

**TABLE 4:** Results and calculated specificities of the commercial methods for the detection of anti-measles IgM antibodies evaluated with the non-measles sera panel (*n* = 187), by subset^*a*^

Method	No. of positive or equivocal results (specificity, %)								95% CI of specificity
	Chikungunya (*n* = 4)	Dengue (*n* = 34)	Parvovirus B19 (*n* = 35)	Roseola (*n* = 38)	Rubella (*n* = 36)	Zika (*n* = 3)	Unknown^*b*^ (*n* = 37)	Total (*n* = 187)	
Enzygnost	0 (100)	0 (100)	7 (80.0)	2 (94.7)	0 (100)	0 (100)	0 (100)	9 (95.2)	90.8–97.6
Euroimmun	1 (75)	0 (100)	6 (82.9)	1 (97.4)	0 (100)	0 (100)	0 (100)	8 (95.7)	91.4–98.0
Euroimmun Nucleoprotein	0 (100)	0 (100)	1 (97.1)	0 (100)	0 (100)	0 (100)	1 (97.3)	2 (98.9)	95.8–99.8
IBL	0 (100)	2 (94.1)	0 (100)	0 (100)	3 (91.7)	1 (66.7)	1 (97.3)	7 (96.3)	92.1–98.3
LIAISON XL	0 (100)	0 (100)	1 (97.1)	0 (100)	0 (100)	0 (100)	0 (100)	1 (99.5^*d*^)	96.6–100
Microimmune	0 (100)	1 (97.1)	0 (100)	2 (94.7)	0 (100)	1 (66.7)	1 (97.3)	5 (97.3)	93.5–99.0
NovaLisa	0 (100)	2 (94.1)	15 (57.1)	2 (94.7)	0 (100)	1 (66.7)	1 (97.3)	21 (88.8^*d*^)	83.1–92.8
Serion (activity calculator)^*c*^	0 (100)	0 (100)	16 (54.3)	2 (94.7)	0 (100)	2 (33.3)	1 (97.3)	21 (88.8^*d*^)	83.1–92.8
Serion (OD range)^*c*^	0 (100)	0 (100)	15 (57.1)	2 (94.7)	0 (100)	2 (33.3)	1 (97.3)	20 (89.3^*d*^)	83.7–93.2
Serion (special case formula)^*c*^	1 (75)	0 (100)	17 (51.4)	3 (92.1)	0 (100)	2 (33.3)	2 (94.6)	25 (86.6^*d*^)	80.7–91.0

a Specimens with equivocal results were counted as positive.

b This panel of sera included fever/rash illness of unknown etiology.

c Three methods of sample result determination, using the single set of optical density data from the test plates, were provided in the manufacturer’s IFU. All three methods were evaluated.

d Significant difference (*P* < 0.05) between the most specific (LIAISON XL) and the least specific methods (Serion, all three result determination methods, and NovaLisa) based on nonoverlapping 95% confidence intervals.

### Assessment of cross-reactivity of measles IgM kits.

All sera in the non-measles sera panel were either IgM positive for other agents that can present with fever and rash symptoms (*n* = 150 sera) or were collected from individuals reported as having fever and rash (*n* = 37) (Table 1). To assess possible cross-reactivity with any specific agent, the number of positive or equivocal results by subset was determined (Table 4). Few false-positive or equivocal results were obtained, with the notable exception of the parvovirus B19 sera, which had a range of 6 to 17 false-positive or equivocal results with the Euroimmun (whole antigen), Enzygnost, NovaLisa, and Serion (all three result determination methods) kits. The specificity, calculated only for the parvovirus B19 sera, ranged from 51.4% (Serion using the special case formula method; 95% CI, 34.3% to 68.3%) to 100% (IBL and Microimmune; 95% CI, 87.7% to 100%). The four kits with the fewest false positives (IBL, Microimmune, LIAISON XL, and Euroimmun NP) had a statistically significantly better specificity than the two kits with the most false positives (NovaLisa and Serion, all three result determination methods).

### Assessment of clinical accuracy of measles IgM kits.

The predetermined classification of the sera as either true measles positive (using the measles case definition) or true measles negative (80% of the sera were confirmed for a fever rash illness of another etiology) (Table 1) was used to determine how accurately each method identified the two classifications of sera (Table 5). When an always wrong approach was used to classify equivocal test results, the reference standard Enzygnost kit had a clinical accuracy of 95.0% (95% CI, 91.2% to 97.3%) and a kappa measure of concordance of 0.858 (95% CI, 0.781 to 0.936). Three methods exceeded the Enzygnost kit, IBL (95.8%), Microimmune (96.2%), and LIAISON XL (96.7%). The two highest-performing kits, LIAISON XL and Microimmune, had a clinical accuracy that was statistically significantly better than that of the method with the lowest accuracy, the Serion kit, specifically using the special case formula method (89.1%; 95% CI, 84.3% to 92.6%). This relationship held true even when equivocal results were classified as presumptive positives; however, the accuracy for the Microimmune kit (97.5%) improved such that it was also significantly higher than that of any of the three result calculation methods for the Serion kit and the NovaLisa kit (Table 5). When a presumptive positive approach was used, the accuracy improved for five of the methods evaluated such that in addition to the Microimmune (97.5%), LIAISON XL (unchanged at 96.7%), and IBL (96.%), the Euroimmun NP (96.2%) method also exceeded the accuracy of the Enzygnost method (95.8%). These five methods all had excellent kappa measures of concordance greater than 0.85. The poorest-performing methods (NovaLisa and Serion) had kappa measures less than 0.8, regardless of how the equivocal results were classified.

**TABLE 5:** Calculated clinical accuracy and kappa statistic for concordance of the commercial methods for the detection of anti-measles IgM antibodies against the predetermined classification of the measles and non-measles sera (*n* = 52 and 187, respectively)^*a*^

Method	Measles specimens with equivocal results counted as negative^*b*^				All specimens with equivocal results counted as positive^*c*^			
	Accuracy (%)	95% CI (%)	Kappa statistic	95% CI	Accuracy (%)	95% CI (%)	Kappa statistic	95% CI
Enzygnost	95.0	91.2–97.3	0.858	0.781–0.936	95.8	92.2–97.9	0.884	0.813–0.954
Euroimmun	92.1	87.7–95.0	0.762	0.660–0.863	94.6	90.7–96.9	0.843	0.761–0.926
Euroimmun Nucleoprotein	93.7	89.7–96.3	0.800	0.704–0.897	96.2	92.7–98.2	0.885	0.812–0.959
IBL	95.8	92.2–97.9	0.880	0.808–0.953	96.2	92.7–98.2	0.893	0.825–0.961
LIAISON XL	96.7^*e*^	93.3–98.4	0.897	0.828–0.967	96.7	93.3–98.4	0.897	0.828–0.967
Microimmune	96.2	92.7–98.2	0.890	0.820–0.960	97.5^*e*^	94.4–99.0	0.928	0.872–0.985
NovaLisa	90.0	85.3–93.3	0.738	0.641–0.835	90.0^*e*^	85.3–93.3	0.738	0.641–0.835
Serion (activity calculator)^*d*^	90.8	86.2–94.0	0.763	0.670–0.855	90.8^*e*^	86.2–94.0	0.763	0.670–0.855
Serion (OD range)^*d*^	91.2	86.7–94.3	0.772	0.681–0.863	91.2^*e*^	86.7–94.3	0.772	0.681–0.863
Serion (special case formula)^*d*^	89.1^*e*^	84.3–92.6	0.726	0.630–0.822	89.1^*e*^	84.3–92.6	0.726	0.630–0.822

a Measles samples with equivocal results were counted as both negative and positive. Non-measles samples with equivocal results were always counted as positive.

b Specimens in the measles sera panel with equivocal results were counted as negative, and specimens in the non-measles sera panel with equivocal results were counted as positive. In this scenario, equivocal results are considered to be “always wrong.”

c All specimens (measles and non-measles) with equivocal results were counted as positive. In this scenario, equivocal results for the measles sera panel were considered correct and incorrect for the non-measles sera panel.

d Three methods of sample result determination, using the single set of optical density data from the test plates, were provided in the manufacturer’s IFU. All three methods were evaluated.

e Significant difference (*P* < 0.05) between the most accurate and the least accurate method(s) based on nonoverlapping 95% confidence intervals. When equivocal results were considered to be “always wrong,” the most accurate method, LIAISON XL, was significantly (*P* < 0.05) better than the Serion kit using the special case formula result determination method. When equivocal results were counted as positive, the most accurate method, Microimmune, was significantly (*P* < 0.05) better than NovaLisa and Serion (all three result determination methods).

### Assessment of sera reactivities.

**FIG 1:**
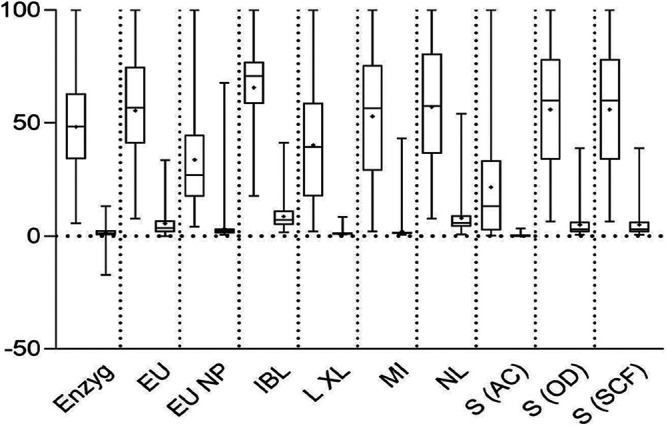
Box plot of output values for each method by sample set, normalized to 100. Each pair corresponds to a test method, with the left plot of each pair representing the output data of the measles samples and the right plot, the non-measles samples. Open bars capture the middle 50% of the values, specifically, from the first quartile (bottom of bar) to the third quartile (top of the bar). The whiskers extend from the minimum to maximum values. The dividing line in the open bars indicates median values. Plus signs (+) indicate mean values. Due to differing test method OD data manipulations generating output values of differing scales, the maximum value for each test kit was set to 100, and all other values were normalized to this value. Enzyg, Enzygnost; EU, Euroimmun; EU NP, Euroimmun NP; L XL, LIAISON XL; MI, Microimmune; NL, NovaLisa; S (AC), Serion (activity calculator result determination method); S (OD), Serion (OD range result determination method); S (SCF), Serion (special case formula result determination method).

All methods generated test results as continuous variables (either the OD value directly or the result of mathematical calculations specified in the IFU) which were then compared to cutoff values to classify the numerical value to a qualitative result. These output values by sample set (measles and non-measles) were compared for each test kit to assess the spread between positive and negative sample sets (Fig. 1). The respective mean values for the measles sera and non-measles sera were calculated, and the fold change was determined between the two sample sets (Table 6). The average fold change was 24, with the NovaLisa kit having the smallest ratio between the measles and non-measles sample sets (7.241) and the Serion kit, using the activity calculator method, having the largest fold change (100.354). These two methods had the worst clinical accuracy values, after the Serion kit using the special case formula (90.0%; 95% CI, 85.3% to 93.3% and 90.8%; 95% CI, 86.2% to 94.0%, respectively). Thus, the spread of the numerical values between true measles positives and negatives was not a good predictor of clinical accuracy.

**TABLE 6:** Mean output values by sample set and fold change between sample set types^*a*^

Method	Measles sera mean output value (min, max)	Non-measles sera mean output value (min, max)	Fold change
Enzygnost	0.678 (0.079, 1.408)	0.022 (–0.241, 0.185)	31.502
Euroimmun	2.382 (0.329, 4.302)	0.241 (0.002, 1.441)	9.883
Euroimmun Nucleoprotein	2.113 (0.261, 6.290)	0.205 (0.036, 4.270)	10.309
IBL	2.826 (0.761, 4.318)	0.373 (0.074, 1.780)	7.563
LIAISON XL	5.211 (0.270, 13.000)	0.151 (0.100, 1.100)	34.556
Microimmune	2.034 (0.080, 3854)	0.078 (0.021, 1.661)	25.909
NovaLisa	37.498 (4.993, 65.877)	5.179 (0.462, 35.606)	7.241
Serion (activity calculator)^*b*^	502.273 (5.395, 2,516.651)	5.005 (0.202, 84.103)	100.354
Serion (OD range)^*b*^	1.940 (0.222, 3.477)	0.174 (0.017, 1.347)	11.141
Serion (special case formula)^*b*^	1.940 (0.222, 3.477)	0.174 (0.017, 1.347)	11.141

a Min, minimum; max. maximum.

b Three methods of sample result determination, using the single set of optical density data from the test plates, were provided in the manufacturer’s IFU. All three methods were evaluated.

## DISCUSSION

Previous extensive evaluations of commercial measles IgM EIA kits resulted in the broad adoption of the Siemens (previously Behring) Enzygnost kit within the WHO global measles and rubella laboratory network (11–13). More recent evaluations have included automated chemiluminescent methods (17–21), but there is still a need for conventional manual EIA methods, which are recommended within the WHO measles and rubella laboratory network (10). Prompted by the discontinuation of the Enzygnost kit (14), six alternative EIA methods and one automated CLIA method were evaluated in this study. The Enzygnost kit was included as a benchmark, not the gold standard, allowing identification of other methods with better performance. The Enzygnost kit had a calculated sensitivity and specificity of 94.2% and 95.2%, respectively, when an always wrong approach was taken to classify the equivocal results (95% CI of 83.1% to 98.5% and 90.8% to 97.6%), in line with previous evaluations that reported an average sensitivity of 93.2% (range, 87.9% to 100%) and specificity of 97.8% (range, 96.7% to 98.7%) (11–13, 17–19). Three of the methods evaluated in this study had an equivalent or better sensitivity (IBL, NovaLisa, and Serion; all three, result determination methods), and five methods had better specificity (LIAISON XL, Euroimmun, Euroimmun NP, IBL, and Microimmune). However, only one kit, from IBL, had both an equivalent sensitivity (94.2%) and improved specificity (96.3%) compared to the Enzygnost kit. No single method had both a sensitivity and specificity of 95% or higher. When it came to overall clinical accuracy, the Enzygnost kit had an excellent accuracy of 95.0% (95% CI, 91.2% to 97.3%; kappa statistic of 0.858), and three methods, LIAISON XL, IBL, and Microimmune, exceeded that benchmark. For the two kits with the lowest clinical accuracy (NovaLisa, 90.0% and Serion, 89.1% to 91.2% depending on the result determination method used), it was their lack of specificity that was the contributor, and in particular with this panel of sera, primarily due to cross-reactivity with sera IgM positive for parvovirus B19.

The presence of parvovirus B19-specific IgM was previously demonstrated to be a source of cross-reactivity for anti-measles IgM detection methods (10, 22–24) and proved to be problematic with some of the methods evaluated in this study. Only the IBL and Microimmune kits did not have any false-positive results with this sample set, while the NovaLisa and Serion kits had the highest numbers of false positives (*n* = 15 to 17). Of the panel of 35 parvovirus-positive sera, 20 (57.1%) resulted in a borderline/equivocal or false-positive result with at least one method, and most of these (*n* = 13) were reactive (equivocal or positive) with at least two methods. Nine sera had false-positive anti-measles IgM results with 2 or 3 methods. This same panel was used to evaluate 8 anti-rubella IgM kits (manuscript in preparation), and many of the same sera that were cross-reactive with the anti-measles IgM kits were also cross-reactive with anti-rubella IgM kits. Of the 20 sera that had a borderline/equivocal or false-positive result with at least one anti-measles IgM method, 17 also had a borderline/equivocal or false-positive result with at least one anti-rubella IgM method. This suggests false-positive results are due to a general cross-reactivity and are not specific to measles IgM kits.

In elimination settings, surveillance systems operate with a focus on sensitivity which, for some tests, can come with a cost of false-positive results, a cause for concern. Furthermore, in these settings, the positive predictive value of IgM serology is greatly diminished. Thus, methods with excellent accuracy are needed. For this evaluation, a high bar was set by taking an “always wrong” approach to classify the equivocal results. However, in practice, to meet enhanced surveillance needs, a suspected measles case with an IgM equivocal result would not be discarded but would instead be reflexed to additional investigation, such as reverse transcriptase PCR (RT-PCR), follow up IgM, or IgG testing. In this scenario, an equivocal IgM result could be considered a presumptive or suspected positive. Using such an approach to classify the equivocal results for the methods in this evaluation improved the clinical accuracy of the best-performing methods as well as the two kits from Euroimmun. The lowest-performing kits (NovaLisa and Serion) remained unchanged with accuracies hovering around 90%.

This study had some limitations. Because residual, anonymized sera were used, information regarding the individual case, such as vaccination history and rash onset date, were unavailable for the measles sera. It is expected that some of the measles cases were confirmed by RT-PCR, but that information was also unavailable. Overall vaccination coverage rates are high in Canada (25), and most cases of measles occur in inadequately vaccinated individuals (26–32). Of the reported measles cases occurring between 2002 and 2013, the years in which these sera were collected, nearly two-thirds of measles cases were unvaccinated (63% of reported cases) and only 17% had received at least one dose of measles vaccine (26). Thus, although vaccination history data were missing from this sample set, it is expected that most cases were unvaccinated.

An additional limitation was that only one lot of each test method was included in the study. While reproducibility was assessed using the results of the external control, it was not evaluated for multiple lots and thus may not be representative of the overall reproducibility of the methods. For the Euroimmun NP kit, however, an additional lot (E181121BK) was evaluated using the entire panel and demonstrated poor concordance with the lot included in this study (data not shown). However, it was noted that the results from one of the three test plates (lot E181121BK) were likely invalid (based on the performance of the external positive control, not the kit controls), and so we are unable to determine if the poor concordance was due to variability between lots.

Detection of measles IgM antibodies remains an important, high-throughput, robust method of providing laboratory confirmation of measles cases, particularly in settings of high disease prevalence. In settings where measles elimination has been achieved, or nearly so, the positive predictive value of IgM serology is greatly diminished, and additional information (including laboratory tests such as RT-PCR) is required for the confirmation of measles cases (10, 24, 33). For this reason, measles virus detection by RT-PCR is often the test of choice for case confirmation. Furthermore, molecular epidemiological methods (RT-PCR and genotyping) allow virological monitoring of the progress and achievement of elimination goals. This study separated out the evaluated methods that had both good sensitivity and specificity (IBL and Microimmune) from those that excelled in one area but not the other (NovaLisa and Serion). Based on overall clinical accuracy, the methods that used IgM capture techniques were the best (LIAISON XL, IBL, and Microimmune) and indeed performed better than the Enzygnost reference standard.
